# Can Diet Prevent Urological Cancers? An Update on Carotenoids as Chemopreventive Agents

**DOI:** 10.3390/nu14071367

**Published:** 2022-03-25

**Authors:** Tomasz Konecki, Aleksandra Juszczak, Marcin Cichocki

**Affiliations:** 1st Department of Urology, Medical University of Lodz, 90-459 Lodz, Poland; aleksandrabeatajuszczak@gmail.com (A.J.); marcin.cichocki@umed.lodz.pl (M.C.)

**Keywords:** prostate cancer, bladder cancer, renal cell carcinoma, carotenoids, α-carotene, β-carotene, β-cryptoxanthin, lutein, lycopene, zeaxanthin

## Abstract

Urological cancers, namely prostate, bladder, kidney, testicular, and penile cancers, are common conditions that constitute almost one-quarter of all malignant diseases in men. Urological cancers tend to affect older individuals, and their development is influenced by modifiable metabolic, behavioral, and environmental risk factors. Phytochemicals may have cancer-fighting properties and protect against cancer development, slow its spread, and reduce the risk of cancer deaths in humans. This paper aims to review the current literature in regard to the effects of carotenoids in reducing urological cancer risk.

## 1. Introduction

As the global population expands and the average life expectancy increases, cancer-related deaths are growing higher. Thus, more efforts are being undertaken to not only treat but prevent certain cancers [1]. Urologic oncology comprises a significant part of the current medical and surgical practice of urologists. Urological cancers, namely prostate, bladder, kidney, testicular, and penile cancers, are common conditions that constitute almost one-quarter of all malignant diseases in men. Urologic cancers tend to affect older individuals, and their development is influenced by modifiable metabolic, behavioral and environmental risk factors. Studies have shown that a significant number of phytochemicals can have a positive impact on the function of human cells [2]. Phytochemicals may have cancer-fighting properties and protect against cancer development, slow its spread, and reduce the risk of deaths due to it in humans [3]. Carotenoids are biologically active phytochemicals that are present in many plant foods. There are two main groups of carotenoids: provitamin A, which is converted into retinol (i.e., α-carotene, β-carotene, and β-cryptoxanthin), and nonprovitamin A, which is not (i.e., lutein, zeaxanthin, and lycopene) [4]. Many researchers have conducted studies to investigate the role of carotenoids in cancer prevention. This paper was aimed at reviewing current literature in regard to effects of carotenoids in reducing urological cancer risk.

A number of urological cancers, namely bladder cancer, prostate cancer, and kidney cancer (BCa, PCa, and RCC, respectively), seem to be at least partly dependent on exposure to environmental factors or dietary habits. Kidney cancer has a well-established risk factor in obesity. It was shown that metabolic syndrome is an independent risk factor for higher pathological grade of kidney cancer [5]. A recently published systematic review and meta-analysis found that patients with metabolic syndrome had a 1.94-fold higher risk of high-stage and a 1.5-fold higher risk of high-grade bladder cancer [6]. In regard to PCa, a prospective population-based Finnish study in a multivariate analysis established metabolic syndrome as an independent risk factor of developing PCa [7], and metabolic syndrome is associated with occurrence of higher-grade, aggressive disease, as shown in the meta-analysis [8]. The three aforementioned urological cancers (BCa, PCa, RCC) are the subject of this review.

The majority of testicular cancer cases are diagnosed between the ages of 20 and 45. Risk factors for the development of testicular cancer are attributable to testicular dysgenesis syndrome and encompass cryptorchidism, hypospadias, impaired spermatogenesis and fertility, and disorders of sex development [9]. There is currently no evidence of any association between testicular cancer and environmental exposure, including diet. This seems to be in line with prevalent hypotheses supporting the prenatal origin of testicular cancer. Penile cancer is and uncommon condition, with an overall incidence of about 1/100,000 in developed countries. About one-third of cases are related to HPV (types 16 and 18) infection-dependent carcinogenesis. Trials looking at the possible role of diet or carotenoids in prevention of this rare tumor are lacking [10].

## 2. Prostate Cancer

**Figure 1:**
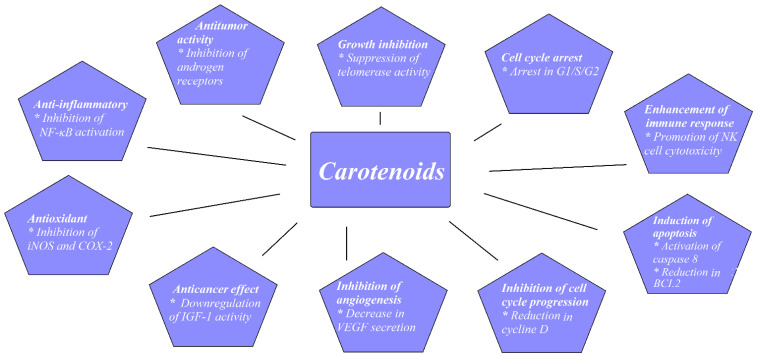
Proposed mechanisms for carotenoids preventing cancer development. Based on figures from Milani et al. [14].

PCa is a common malignancy among men worldwide. In 2018, PCa was reported as the second most common noncutaneous cancer in men worldwide (an estimated 1.3 million new cases) and the fifth most common cause of cancer death in men. There is wide geographical diversity in its prevalence, with age-standardized rates (ASR) per 100,000 of 97.2 in the United States and less than 20 in Asia and developing countries [11]. The most common pathological type of PCa is acinar adenocarcinoma, which accounts for more than 99% of all prostate tumors [12]. Family history of PCa, older age, and race are the only undisputed risk factors for PCa development. Asian men have low PCa risk compared with men from the Western world. However, when Japanese men moved from Japan to California, their risk of PCa rose, approaching that of American men, which implied a role of dietary or environmental factors, possibly including dietary carotenoid intake as a contributing factor [13]. There have been a few proposed mechanisms through which carotenoids may act to reduce cancer development, as shown in Figure 1.

### 2.1. Experimental Studies

The inhibition of PCa cell growth has been the subject of a number of experimental studies performed on the PCa cell cultures LNCap, DU145, and PC-3. The LNCaP cell line comes from a lymph node metastasis of human PCa; DU145 cells are derived from a brain metastasis of primary PCa; and the PC-3 cell line was established from a PCa bone metastasis from a 62-year-old male Caucasian. The growth-inhibitory effect of lycopene was compared with that of one of the main chemotherapeutic agents used to treat metastatic PCa, docetaxel. PCa cell growth was inhibited by 19–54% with docetaxel and by 5–24% with lycopene. A synergic effect of 78% growth reduction was also shown when combining docetaxel with lycopene [15,16]. A dose-dependent reduction in PCa cellular growth was shown with rising lycopene concentrations of up to 10 µM [17,18]. It may be suggested that the efficacy of lycopene depends on its source of origin. Lycopene extracts from algae (marine Chlorella) and from tomatoes were compared at concentrations of 20 and 50 µM. PCa cells’ viability was 10–18% lower after treatment with algae lycopene than after treatment with tomato lycopene at the same concentration, showing the better antiproliferative and apoptotic effect of algal lycopene [19]. Similarly, extracts from tomato paste, tomato sauce, ketchup, and tomato extract were compared on PCa cells that were obtained and cultured from prostates of patients submitted to radical prostatectomy. Cell growth reduction was highest for tomato paste, and then for ketchup, tomato sauce, and extract, with reductions in cellular growth of 54%, 51%, 47%, and 44%, respectively [20]. One must be cautious when interpreting cell-culture-based studies because there are differences between the concentrations of carotenoids in the body and those used in cell culture, with the latter usually being significantly higher. Concentrations of 1 µM and 0.5 µM of lycopene reduced cell proliferation of LNCaP cells; however, there was no effect observed at 0.25 µM [21]. Other studies have suggested that higher lycopene concentrations of around 1–1.25 µM lycopene are necessary for the antiproliferative effect [22]. Postmortem human studies showed that carotenoid concentrations in serum and individual organs were similar and that there was a correlation between intake and tissue/blood levels of carotenoids. The serum concentration of lycopene in plasma was around 740 nmol/kg, and that in prostate tissue was around 700 nmol/kg. These physiological concentrations may be at the edge of those that showed an antiproliferative effect in experimental studies.

Palozza et al. showed in an in vitro study on PCa cells that β-carotene acted as a growth-inhibitory agent by inhibiting AKT phosphorylation, which led to the increased expression of c-MYC and the activity of caspases. In the end, apoptosis was stimulated [23].

Furthermore, in another experimental animal study, Yang et al. subcutaneously implanted androgen-independent PCa (PC-3) cells in mice. Both lycopene and β-carotene, when supplemented to the mice, significantly inhibited tumor growth [24].

There have been few interesting studies, however, that have examined the impact of lycopene on PCa cells’ ability to spread and metastasize. Using PC3 and DU145 PCa cell lines, Elgass et al. showed that the adhesion of PCa cells to a basement membrane was significantly reduced at lycopene concentrations higher than 1.15 µM. There was also a reduction in PCa cellular motility and migration of 40% and 58% for PC3 cells and DU145 cells, respectively [25]. Some proteins known to be responsible for controlling the adhesion and migration of PC (namely intercellular adherence molecule 1 (ICAM1) and MMP9) were downregulated in PC-3 cell lines treated with tomato extract. These results indicate the ability of LC to diminish PCa’s metastatic abilities to some degree. The expression of proteins such as intracellular ICAM 1 and MMP9 was influenced by tomato extract and downregulated in PC3 cell lines [26]. This may suggest the potential ability of lycopene to reduce PCa spread.

### 2.2. Epidemiological Studies

It has been suggested that saturated fat consumption is linked to not only PCa incidence but PCa progression and mortality risk [27]. One of the key oncogenes in PCa tumorigenesis is c-MYC. It has been shown that increased fat consumption results in amplifying the MYC-dependent transcriptional cascade [28]. Blood lipids, and especially high lipoprotein A concentrations, seem to be associated with increased PCa risk and advanced disease [29], and lipoprotein A is known to promote inflammation. On the other hand, high levels of chronic inflammatory markers are associated with high-risk, aggressive PCa [30]. The well-established role of carotenoids as scavengers of reactive oxygen species and anti-inflammatory agents might contribute to PCa risk reduction [31].

High concentrations of carotenoids were identified in some vegetables consumed as part of a traditional Mediterranean diet [32]. There have been a few prospective cohort studies that found no protective association between Mediterranean diet prior to diagnosis and incidence of lethal or advanced PCa. However, an interesting observation was made that greater adherence to Mediterranean diet after the diagnosis of nonmetastatic PCa lead to 22% post-treatment reduction in overall mortality [33].

Hoang et al., in a case–control study with 652 participants, showed that PCa patients consumed significantly lower levels of carotenoids than controls (*p* < 0.05). In comparison with the highest versus the lowest tertile of lycopene or carrot and tomato intake, the odds ratios for PCa were 0.46 and 0.39, respectively. These correlations were true for low-, medium-, and high-grade PCa [34].

Antwi et al. found that higher dietary lycopene intake was associated with a decreased risk of having aggressive PCa. The odds ratio (OR) was 0.55 in the highest versus the lowest tertile after adjustment for multiple covariates. There were three groups of PCa defined in the study: highly aggressive PCa (Gleason sum ≥ 8 or PSA > 20 ng/mL or Gleason sum ≥ 7 and clinical stages T3–T4), low aggressive (Gleason sum < 7 and clinical stages T1–T2 and PSA < 10 ng/mL), and intermediate aggressive (all others). They also found that African-Americans, but not European-Americans, with higher β-cryptoxanthin dietary intake had 45% lower odds of aggressive PCa [4].

Because up to 80% of people develop some form of indolent PCa in their lives, it is more important for the urological community to look for factors associated with high-grade, clinically significant disease than to concentrate on any PCa. Cases are dominated by low-grade Gleason 6, mostly clinically nonsignificant disease. It seems that studies that included PCa patients diagnosed before the PSA-era with less indolent cancers reported more pronounced associations between carotenoid intake and PCa risk.

There seems to be more consistent evidence for a modest protective role for carotenoids against PCa progression than general incidence or initiation of the disease itself [35,36,37].

In general, the most robust evidence in the literature regarding the role of carotenoids in PCa has been associated with lycopene as a potent antioxidant. There have been three meta-analyses published in the literature on this topic, which are summarized in Table 1.

α- and β-carotene can be found in carrots, the consumption of which is common worldwide [41]. In a meta-analysis regarding carrot consumption and risk of PCa, the authors found a significantly decreased risk of PCa associated with the intake of carrots [42]. They found that increasing carrot consumption by one serving per week or 10 g per day was associated with 5% and 4% decreases in the risk of PCa, respectively. It has been shown that BMI might be a factor associated with an increased risk of advanced PCa and a higher risk of PCa death. Few studies controlled for this factor. However, pooled analysis of studies included in the meta-analysis that controlled for BMI showed similar results of significant PCa risk reduction associated with carrot consumption.

There is an issue with the accuracy with which diet records and food frequency questionnaires can accurately estimate lycopene intake in the long run due, for example, to significant variations of lycopene concentration in foods.

Some researchers examined the relationship between the concentration of circulating lycopene and the risk of PCa.

Nordstrom et al. studied a group of 559 men undergoing radical prostatectomy due to nonmetastatic PCa They examined the risk of high-risk PCa, defined as Gleason sum ≥ 8, PSA level at diagnosis ≥ 20 ng/mL, or T-stage ≥ T3, in relation to circulating carotenoids. They found a significant association, with OR between 0.31 and 0.55, between circulating levels of α-carotene, β-carotene, lycopene, and total carotenoids and high-risk PCa [43].

However, in general, studies aimed at the association between carotenoid concentration in plasma and PCa risk have generated equivocal results.

Although there have been a number of studies suggesting that carotenoids may play a protective role in PCa incidence, the overall evidence remains inconclusive.

## 3. Bladder Cancer

Bladder cancer (BCa) is among the ten most common cancers around the world, with about 430,000 cases diagnosed per year [44]. Smoking is by far the greatest risk factor for BCa, being responsible for around 50% of cases. Current versus never smokers have pooled relative risk (RR) of around 3.5 [45]. Occupational exposure to carcinogens has been widely studied and accounts for around 5% of the attributable risk of BCa [46]. As many cases of BCa are caused by exogenous chemicals, antioxidants, such as carotenoids, could potentially play an important protective role.

### 3.1. Case–Control Studies

Vena et al. conducted a case–control study and showed that carotenoid consumption was associated with decreased risk of BCa, with an OR of 0.45 for the highest versus the lowest consumption quartile. They performed a multivariate analysis and adjusted for multiple covariates, such as kilocalories, age, education, cigarette smoking, and total fluid consumption [47]. The protective effect of carotenoids was also observed by Castelao et al. in a population-based case–control study with 1592 BCa patients. BCa risk was inversely correlated with total carotenoid consumption (*p* = 0.004) [48].

Brinkman et al. summarized the results of a population-based case–control study conducted in New Hampshire, United States. No statistically significant influence of total carotenoid consumption on BCa incidence was observed, nor for individual carotenoids, α-carotene, b-carotene, β-cryptoxanthin, lycopene, or lutein/zeaxanthin. Within the group of heavy smokers, >20 cigarettes a day, an inverse association of borderline statistical significance was observed for the highest consumption of total carotenoids (OR: 0.62; 95% CI: 0.36–1.09) [49]. Similarly, Wakai et al. did not show a protective impact of carotenoid consumption in their case–control study, although a lower risk of BCa was observed in heavier smokers (cumulative smoking dose < 400 versus ≥400 cigarettes per year) [50]. Shabath et al., apart from the overall protective effect of carotenoid intake, found that compared with the reference OR of 1.0 in a group of never smokers with a high total carotenoid intake, current smokers with a low total carotenoid intake had a significant 4.3-fold increased risk (95% CI, 1.56–12.09). The ORs were less pronounced for current smokers with high carotenoid intake and for former smokers with low carotenoid intake (ORs of 3.04 and 2.3, respectively) [51]. The above observations could suggest that the protective effect of carotenoid consumption may be more pronounced in heavier smokers, working as a sort of counterbalance against the negative impact of smoking.

In a case–control study of 856 patients with newly diagnosed urothelial cell carcinoma, 405 cases were classified as aggressive cancer. Total plasma carotenoid concentration showed an inverse association with urothelial cell cancer risk, and β-carotene was inversely correlated with aggressive but not with nonaggressive UCC [52].

Serum levels of lycopene and B-carotene were not different between cases and controls in a nested case–control trial conducted in Washington County. The prospective cohort consisted of 20,305 individuals who were followed for 12 years, with 35 BCa cases diagnosed [53]. In another nested case–control study with 111 BCa patients, there were statistically significant inverse linear trends in risk for α-carotene, β-carotene, zeaxanthin, β-cryptoxanthin, and total carotenoids; however, these were no longer significant after adjustment for pack-years of cigarette smoking [54]. A number of case–control studies showed no significant impact of carotenoid consumption on BCa incidence [49,55,56,57].

### 3.2. β-carotene Supplementation Studies

Few studies have examined β-carotene supplementation and risk of BCa.

No protective effect was found in the Alpha-Tocopherol, Beta-Carotene Cancer Prevention (ATBC) interventional prospective study, in which participants (*n* = 29,133) were randomly assigned to one of four groups, receiving α-tocopherol (50 mg), β-carotene (20 mg), both agents, or a placebo, for a long-term median period of 6.1 years [58].

Roswall et al. reported the results of the Danish Diet, Cancer, and Health Study, a prospective cohort study. They found a significant association between dietary β-carotene intake and risk of BCa that was not observed for supplemental intake of carotene. The subgroup analysis conducted in groups stratified by smoking status (never, former, current smokers) revealed that the protective effect of dietary carotene intake was highest among current smokers, with an incidence rate ratio of 0.67 [59].

Hotaling et al. reported no significant correlation between β-carotene supplementation and BCa risk in a cohort study of 77,050 participants [60].

### 3.3. Cohort Studies

Recently, a large population study of prostate, lung, colorectal, and ovarian cancer (PLCO) screening (99,650 individuals) was used to evaluate the association between dietary tomato or lycopene consumption and BCa. Seven hundred and seventy-four cases of BCa were observed after a median of 12.5 years of follow-up. Dietary intakes of raw tomatoes, tomato ketchup, tomato salsa, tomato juice, and lycopene were not associated with BCa risk [61].

Wu et al. published a large meta-analysis with 516,740 patients involved. When they compared the highest with the lowest categories of total carotenoid intake and concentrations of circulating carotenoids, a trend of protective impact was observed, although the results were not significant with RR = 0.88 (0.76–1.03) and RR = 0.36 (0.12–1.07). Significant heterogeneity was found among studies of total carotenoid intake and among studies of circulating carotenoid concentrations. On dose–response analysis of individual carotenoids (*α*-carotene, β-carotene, β-cryptoxanthin, zeaxanthin, and lycopene), statistically significant inverse linear associations were present for dietary intake of β-cryptoxanthin (RR = 0.58; 95% CI: 0.36, 0.94) and circulating concentrations of *α*-carotene (RR = 0.24; 95% CI: 0.08, 0.67), β-carotene (RR = 0.73; 95% CI: 0.57, 0.94), and zeaxanthin (RR = 0.44; 95% CI: 0.28,0.67) [62]. A recently conducted meta-analysis that studied the association between BCa risk and dietary habits showed that the Mediterranean diet had a protective effect on BCa risk [63].

The main observational studies from the last two decades are summarized in Table 2.

Although multiple large epidemiological studies were positive in showing that lycopene could reduce the risk of PCa, there has been no consistent evidence that consumption of lycopene or tomatoes has a similar effect in BCa. Future large prospective studies could possibly bring more definitive conclusions on the effects of carotenoid intake on the risk of BCa. 

## 4. Kidney Cancer

Kidney cancer is a broad term used to describe a heterogeneous group of tumors from a histological point of view. Kidney cancer can develop from renal parenchyma or the renal collecting system. Renal cell carcinoma (RCC) arises from renal parenchyma epithelial cells and accounts for approximately 90% of kidney cancer cases. RCC accounts for around 3% of all cancers, with the highest incidence reported in Western countries [11]. The most common histological type of RCC is clear cell RCC, which represents ~80% of RCCs. Among the other histological subtypes of kidney cancer, papillary (~10%) and chromophobe (~5%) are the most common. Clear cell and papillary RCCs stem from the epithelial cells of the proximal tubule. Chromophobe RCC is thought to arise from the epithelium of the collecting tubule [69]. Well-established risk factors include smoking, obesity, hypertension, and diabetes [70]. Carotenoids, as potent antioxidants, may influence RCC risk.

Recently, Sahin et al. conducted a very interesting experimental study to examine the role of a lycopene-rich diet in the development of RCC in the tuberous sclerosis 2 (TSC2) mutant Eker rat model. Eker rats develop spontaneous renal tumors and leiomyoma, which may be due to tuberous sclerosis 2 (TSC2) mutation resulting in the activation of the mammalian target of the rapamycin (mTOR) pathway. Eker rats received 0, 100, or 200 mg/kg of lycopene as part of their diet. After 18 months, the mean numbers of renal carcinomas were statistically significantly decreased in the lycopene-treated rats (*p* < 0.008) when compared with the untreated controls. Tumor numbers decreased linearly as the daily lycopene increased from 0 to 200, suggesting a role of lycopene in the prevention of RCC [71].

Bock et al. recently published the results of the U.S. Kidney Cancer Study, which was a population-based case–control study. Clear, significant inverse associations with RCC risk were found for α-carotene, β-carotene, lutein, zeaxanthin, and lycopene after adjusting for all variables [72].

In another case–control study conducted on 1138 histologically confirmed cases of RCC, Hu et al. showed a significant reduction in RCC risk, with ORs of 0.74 and 0.77 for the highest versus the lowest quartiles for β-carotene and lutein/zeaxanthin. This relationship was more pronounced in women, obese, and ever smoking individuals [73]. Two other case–control studies supported a significant protective influence of carotenoids on RCC risk [74,75]. However, a case–control study by Bosetti et al. did not show any protective impact of carotenoid intake and RCC risk [76].

The Women’s Health Initiative (WHI) was a prospective cohort study that included 96,196 postmenopausal women who were followed for up to 12 years.

There were 240 cases of RCC diagnosed during follow-up. Lycopene intake was inversely correlated with RCC risk. Individuals with the highest quartile of intake had a 39% lower risk of RCC compared with those in the lowest quartile. Interestingly, none of the other evaluated carotenoids (dietary β-carotene, α-carotene, β-cryptoxanthin, and lutein plus zeaxanthin) showed any significant association [77].

On the other hand, a prospective cohort study with 88,759 women and 47,828 men did not show a significant influence of lycopene on RCC risk, although it did find significant correlations for β-carotene, α-carotene, β-cryptoxanthin, and lutein plus zeaxanthin [78]. It was shown in the ATBC prospective study that neither lycopene nor other carotenoid intake was correlated with RCC risk [79], and this confirmed other cohort studies that showed null associations between carotenoids and RCC [80,81].

In 2009, Lee published a pooled cohort analysis of 13 prospective studies that included 1478 cases of RCC among 530,469 women and 244,483 men, who were followed for up to 7 to 20 years. It showed that increased vegetable and fruit consumption was associated with a decreased risk of RCC. Among the specific carotenoids they observed, there was an 18% lower risk of RCC when comparing the highest and lowest quintiles of β-carotene intake. There was a similar trend for other carotenoids (α-carotene, β-cryptoxanthin, and lutein/zeaxanthin) [82].

Zhang et al. recently published a meta-analysis that encompassed 19 observational studies (5 cohort and 14 case–control) with 10,215 RCC cases, summarizing current evidence from epidemiological data on the topic of fruit and vegetables and RCC risk. Although they did not examine carotenoid intake in their work, they found significant reductions in RCC risk for the highest versus the lowest intake of vegetables and fruit (RR = 0.73 and 0.86 respectively) [83]. It can only be speculated that carotenoids were at least partially responsible for the observed effect.

## 5. Conclusions

In summary, numerous studies have suggested that carotenoids may have a protective role in the development of urological cancers and that a diet rich in carotenoids may act as a chemoprevention. The most robust body of literature addresses the potential protective benefit of carotenoids against prostate cancer. In particular, lycopene, as one of the most effective oxygen radical-scavenging agents among the carotenoids, is thought to decrease PCa risk. In BCa and RCC, many observational studies have confirmed the preventive potential of carotenoids, which sounds intuitively logical, as risk factors for urological cancers seem to have an environmental background. However, the findings from many observational studies would require confirmation in large randomized clinical trials of carotenoid supplementation in order to finally elucidate the problem and be able to elaborate guidelines and recommendations. Because the existing evidence in all of the urological cancers is not strong enough to recommend the use of carotenoids in disease prevention, the joint statement of World Cancer Research Fund International/American Institute for Cancer Research still advocates the consumption of fruit and vegetables for cancer prevention and cancer survivors [11].

## Figures and Tables

**Table 1:** Characteristics of meta-analyses assessing carotenoids’ effect on prostate cancer risk.

Author	Number of Studies	Measure	Results
Chen et al., 2013 [38]	17 studies; 6 cohort, 11 NCC	Effect of: Lycopene intake on risk of PCa Serum lycopene on risk of PCa Lycopene intake on risk of advanced PCa Serum lycopene on risk of advanced PCa	Not significant Not significant Not significant Not significant
Wang et al., 2015 [39]	34 studies; 10 cohort 11 NCC 13 CC	Effect of: α-carotene, β-carotene, lycopene intake on PCa α-carotene, β-carotene, lycopene blood concentrations on PCa	Significant inverse association between dietary α-carotene intake and PCa (RR: 0.81; CI: 0.76–0.99). No difference for β-carotene and lycopene intake. Only blood levels of lycopene were significantly associated with reduced PCa risk (RR: 0.81; CI: 0.69–0.96)
Rowles et al., 2017 [40]	42 studies; 19 CC 13 NCC 8 cohort 2 case-cohort	Effect of: Lycopene intake on PCa Lycopene circulating concentrations on PCa	Dietary intake (RR = 0.88, CI: 0.78–0.98) and circulating concentrations (RR = 0.88, CI: 0.79–0.98) of lycopene were significantly associated with reduced PCa risk

CC, case–control study; NCC, nested case–control study; CI, confidence interval; RR, relative risk.

**Table 2:** Characteristics of epidemiological studies assessing carotenoids’ effect on bladder cancer risk.

Study	Study Design	Location/Period	Sex	Cases, *n*	Controls, *n*	Participants, *n*	Measure	Results
Park et al., 2013 [64]	Cohort	United States/1993–2007	M/F	581	—	185,885	Intake of: α-carotene; β-carotene; lycopene; β-cryptoxanthin lutein	α-carotene RR = 0.52 (CI: 0.32–0.83), β-carotene RR = 0.55 (CI: 0.35–0.89) and β-cryptoxanthin RR = 0.47 (CI: 0.28–0.77) led to significantly lower risk of bladder cancer in women
Wu et al., 2012 [65]	PCC	United States/2001–2004	M/F	1087	1266	2353	Intake of: α-carotene β-carotene	No significant difference for α-carotene or β-carotene
Ros et al., 2012 [52]	NCC	10 European countries/1992–2005	M/F	856	856	1712	Plasma concentration of: Total carotenoids; α-carotene; β-carotene; β-cryptoxanthin; lycopene; zeaxanthin and lutein	Plasma β-carotene inversely associated with aggressive BCa (RR: 0.51; CI: 0.30- 0.88)
Brinkman et al., 2010 [49]	PCC	United States/2000–2003	M/F	322	239	561	Intake of: total carotenoids α-carotene β-carotene β-cryptoxanthin lycopene lutein	Total intake of carotenoids inversely related to BCa risk in older men (OR: 0.59; CI: 0.35–0.99)
Hotaling et al., 2011 [60]	Cohort	United States/2000–2005	M/F	319	-	77,050	Supplementation: β-carotene	No significant association with BCa risk
Roswall et al., 2009 [59]	Cohort	Denmark/1993–2006	M/F	322		55,557	β-carotene total intake β-carotene dietary intake β-carotene supplementation	Significantly lower risk of BCa with dietary β-carotene consumption (RR: 0.82; CI: 0.69–0.98) and a borderline significantly lower risk with β-carotene (RR: 0.85; CI: 0.73–1.00)
García-Closas et al., 2007 [66]	HCC	United States/1998–2001	M/F	912	873	1785	Intake of: total carotenoids	No significant association with BCa risk
Hung et al., 2006 [67]	HCC	United States/1993–1997	M/F	84	173	257	Plasma concentration of: α-carotene β-carotene β-cryptoxanthin lycopene lutein zeaxanthin	Significant for α-carotene (OR = 0.22; CI: 0.05–0.92) luteine (OR = 0.42; CI 0.18–1.00), zeaxanthin (OR = 0.16; CI: 0.02–1.06), lycopene (OR = 0.94; CI: 0.89–0.99), and β-cryptoxanthin (OR = 0.90; CI: 0.81–1.00)
Holick et al., 2005 [68]	Cohort	United States/1980–2000	F	237		88,796	α-carotene β-carotene β-cryptoxanthin lutein/zeaxanthin lycopene	No significant association with BCa risk
Castelao et al., 2004 [48]	PCC	United States/1987–1996	M/F	1592	1592	3184	Total carotenoids α-carotene β-carotene β-cryptoxanthin lutein/zeaxanthin lycopene	BCa risk inversely associated with intake of total carotenoids, α-carotene, β-carotene, lutein/zeaxanthin, and lycopene

F, female; HCC, hospital-based case–control study; M, male; NCC, nested case–control study; PCC, population-based case–control study; OR, odds ratio; CI, confidence interval.

## References

[1] Siegel R.L., Miller K.D., Jemal A. (2016). Cancer Statistics, 2016. CA A Cancer J. Clin..

[2] Budisan L., Gulei D., Zanoaga O.M., Irimie A.I., Sergiu C., Braicu C., Gherman C.D., Berindan-Neagoe I. (2017). Dietary Intervention by Phytochemicals and Their Role in Modulating Coding and Non-Coding Genes in Cancer. Int. J. Mol. Sci..

[3] Yoo S., Kim K., Nam H., Lee D. (2018). Discovering Health Benefits of Phytochemicals with Integrated Analysis of the Molecular Network, Chemical Properties and Ethnopharmacological Evidence. Nutrients.

[4] Antwi S.O., Steck S.E., Su L.J., Hebert J.R., Zhang H., Craft N.E., Fontham E.T.H., Smith G.J., Bensen J.T., Mohler J.L. (2016). Carotenoid Intake and Adipose Tissue Carotenoid Levels in Relation to Prostate Cancer Aggressiveness among African-American and European-American Men in the North Carolina–Louisiana Prostate Cancer Project (PCaP). Prostate.

[5] Zhang Q., Chen P., Tian R., He J., Han Q., Fan L. (2022). Metabolic Syndrome Is an Independent Risk Factor for Fuhrman Grade and TNM Stage of Renal Clear Cell Carcinoma. Int. J. Gen. Med..

[6] Feng D., Song P., Yang Y., Wei W., Li L. (2021). Is Metabolic Syndrome Associated with High Tumor Grade and Stage of Bladder Cancer: A Systematic Review and Meta-Analysis. Transl. Cancer Res..

[7] Grundmark B., Garmo H., Loda M., Busch C., Holmberg L., Zethelius B. (2010). The Metabolic Syndrome and the Risk of Prostate Cancer under Competing Risks of Death from Other Causes. Cancer Epidemiol. Biomark. Prev..

[8] Gacci M., de Nunzio C., Sebastianelli A., Salvi M., Vignozzi L., Tubaro A., Morgia G., Serni S. (2017). Meta-Analysis of Metabolic Syndrome and Prostate Cancer. Prostate Cancer Prostatic Dis..

[9] del Giudice F., Kasman A.M., de Berardinis E., Busetto G.M., Belladelli F., Eisenberg M.L. (2020). Association between Male Infertility and Male-Specific Malignancies: Systematic Review and Meta-Analysis of Population-Based Retrospective Cohort Studies. Fertil. Steril..

[10] Hartwig S., Syrjänen S., Dominiak-Felden G., Brotons M., Castellsagué X. (2011). Estimation of the Epidemiological Burden of Human Papillomavirus-Related Cancers and Non-Malignant Diseases in Men in Europe: A Review. BMC Cancer.

[11] World Health Organization (2020). Regional Office for Europe. World Cancer Report: Cancer Research for Cancer Development.

[12] Marcus D.M., Goodman M., Jani A.B., Osunkoya A.O., Rossi P.J. (2012). A Comprehensive Review of Incidence and Survival in Patients with Rare Histological Variants of Prostate Cancer in the United States from 1973 to 2008. Prostate Cancer Prostatic Dis..

[13] Kimura T., Sato S., Takahashi H., Egawa S. (2021). Global Trends of Latent Prostate Cancer in Autopsy Studies. Cancers.

[14] Bolhassani A., Milani A., Basirnejad M., Shahbazi S. (2017). Carotenoids: Biochemistry, Pharmacology and Treatment Correspondence Associate Professor LINKED ARTICLES. Br. J. Pharmacol..

[15] Tang Y., Parmakhtiar B., Simoneau A.R., Xie J., Fruehauf J., Lilly M., Zi X. (2011). Lycopene Enhances Docetaxel’s Effect in Castration-Resistant Prostate Cancer Associated with Insulin-like Growth Factor I Receptor Levels. Neoplasia.

[16] Gong X., Marisiddaiah R., Zaripheh S., Wiener D., Rubin L.P. (2016). Mitochondrial β-Carotene 9, 10 Oxygenase Modulates Prostate Cancer Growth via NF-ΚB Inhibition: A Lycopene-Independent Function Running Title: BCO2 Inhibits NF-ΚB Signaling in Prostate Cancer. Mol. Cancer Res..

[17] Yang C.M., Lu Y.L., Chen H.Y., Hu M.L. (2012). Lycopene and the LXRα Agonist T0901317 Synergistically Inhibit the Proliferation of Androgen-Independent Prostate Cancer Cells via the PPARγ-LXRα-ABCA1 Pathway. J. Nutr. Biochem..

[18] Yang C.M., Lu I.H., Chen H.Y., Hu M.L. (2012). Lycopene Inhibits the Proliferation of Androgen-Dependent Human Prostate Tumor Cells through Activation of PPARγ-LXRα-ABCA1 Pathway. J. Nutr. Biochem..

[19] Renju G.L., Muraleedhara Kurup G., Bandugula V.R. (2014). Effect of Lycopene Isolated from Chlorella Marina on Proliferation and Apoptosis in Human Prostate Cancer Cell Line PC-3. Tumor Biol..

[20] Soares N.d.C.P., Machado C.L., Trindade B.B., Lima I.C.d.C., Gimba E.R.P., Teodoro A.J., Takiya C., Borojevic R. (2017). Lycopene Extracts from Different Tomato-Based Food Products Induce Apoptosis in Cultured Human Primary Prostate Cancer Cells and Regulate TP53, Bax and Bcl-2 Transcript Expression. Asian Pac. J. Cancer Prev..

[21] Ivanov N.I., Cowell S.P., Brown P., Rennie P.S., Guns E.S., Cox M.E. (2007). Lycopene Differentially Induces Quiescence and Apoptosis in Androgen-Responsive and -Independent Prostate Cancer Cell Lines. Clin. Nutr..

[22] Assar E.A., Vidalle M.C., Chopra M., Hafizi S. (2016). Lycopene Acts through Inhibition of IκB Kinase to Suppress NF-ΚB Signaling in Human Prostate and Breast Cancer Cells. Tumor Biol..

[23] Palozza P., Sestito R., Picci N., Lanza P., Monego G., Ranelletti F.O. (2008). The Sensitivity to β-Carotene Growth-Inhibitory and Proapoptotic Effects Is Regulated by Caveolin-1 Expression in Human Colon and Prostate Cancer Cells. Carcinogenesis.

[24] Yang C.M., Yen Y.T., Huang C.S., Hu M.L. (2011). Growth Inhibitory Efficacy of Lycopene and β-Carotene against Androgen-Independent Prostate Tumor Cells Xenografted in Nude Mice. Mol. Nutr. Food Res..

[25] Elgass S., Cooper A., Chopra M. (2014). Lycopene Treatment of Prostate Cancer Cell Lines Inhibits Adhesion and Migration Properties of the Cells. Int. J. Med. Sci..

[26] Kolberg M., Pedersen S., Bastani N.E., Carlsen H., Blomhoff R., Paur I. (2015). Tomato Paste Alters NF-B and Cancer-Related MRNA Expression in Prostate Cancer Cells, Xenografts, and Xenograft Microenvironment. Nutr. Cancer.

[27] van Blarigan E.L., Kenfield S.A., Yang M., Sesso H.D., Ma J., Stampfer M.J., Chan J.M., Chavarro J.E. (2015). Fat Intake after Prostate Cancer Diagnosis and Mortality in the Physicians’ Health Study. Cancer Causes Control.

[28] Labbé D.P., Zadra G., Yang M., Reyes J.M., Lin C.Y., Cacciatore S., Ebot E.M., Creech A.L., Giunchi F., Fiorentino M. (2019). High-Fat Diet Fuels Prostate Cancer Progression by Rewiring the Metabolome and Amplifying the MYC Program. Nat. Commun..

[29] Ioannidou A., Watts E.L., Perez-Cornago A., Platz E.A., Mills I.G., Key T.J., Travis R.C., Tsilidis K.K., Zuber V. (2022). The Relationship between Lipoprotein A and Other Lipids with Prostate Cancer Risk: A Multivariable Mendelian Randomisation Study. PLoS Med..

[30] Cacciatore S., Wium M., Licari C., Ajayi-Smith A., Masieri L., Anderson C., Salukazana A.S., Kaestner L., Carini M., Carbone G.M. (2021). Inflammatory Metabolic Profile of South African Patients with Prostate Cancer. Cancer Metab..

[31] Kawata A., Murakami Y., Suzuki S., Fujisawa S. (2018). Anti-Inflammatory Activity of β-Carotene, Lycopene and Tri-n-Butylborane, a Scavenger of Reactive Oxygen Species. Vivo.

[32] Su Q., Rowley K.G., Itsiopoulos C., O’dea K. (2002). ORIGINAL COMMUNICATION Identification and Quantitation of Major Carotenoids in Selected Components of the Mediterranean Diet: Green Leafy Vegetables, Figs and Olive Oil. Eur. J. Clin. Nutr..

[33] Kenfield S.A., Dupre N., Richman E.L., Stampfer M.J., Chan J.M., Giovannucci E.L. (2014). Mediterranean Diet and Prostate Cancer Risk and Mortality in the Health Professionals Follow-up Study. Eur. Urol..

[34] van Hoang D., Pham N.M., Lee A.H., Tran D.N., Binns C.W. (2018). Dietary Carotenoid Intakes and Prostate Cancer Risk: A Case-Control Study from Vietnam. Nutrients.

[35] Zu K., Mucci L., Rosner B.A., Clinton S.K., Loda M., Stampfer M.J., Giovannucci E. (2014). Dietary Lycopene, Angiogenesis, and Prostate Cancer: A Prospective Study in the Prostate-Specific Antigen Era. J. Natl. Cancer Inst..

[36] Key T.J., Appleby P.N., Allen N.E., Travis R.C., Roddam A.W., Jenab M., Egevad L., Tjønneland A., Johnsen N.F., Overvad K. (2007). Plasma Carotenoids, Retinol, and Tocopherols and the Risk of Prostate Cancer in the European Prospective Investigation into Cancer and Nutrition study. Am. J. Clin. Nutr..

[37] Giovannucci E. (2011). Commentary: Serum Lycopene and Prostate Cancer Progression: A Re-Consideration of Findings from the Prostate Cancer Prevention Trial. Cancer Causes Control.

[38] Chen J., Song Y., Zhang L. (2013). Lycopene/Tomato Consumption and the Risk of Prostate Cancer: A Systemic Review and Meta-Analysis of Prospective Studies. J. Nutr. Sci. Vitaminol. (Tokyo).

[39] Wang Y., Cui R., Xiao Y., Fang J., Xu Q. (2015). Effect of Carotene and Lycopene on the Risk of Prostate Cancer: A Systematic Review and Dose-Response Meta-Analysis of Observational Studies. PLoS ONE.

[40] Rowles J.L., Ranard K.M., Smith J.W., An R., Erdman J.W. (2017). Increased Dietary and Circulating Lycopene Are Associated with Reduced Prostate Cancer Risk: A Systematic Review and Meta-Analysis. Prostate Cancer Prostatic Dis..

[41] Li C., Ford E.S., Zhao G., Balluz L.S., Giles W.H., Liu S. (2011). Serum α-Carotene Concentrations and Risk of Death among US Adults: The Third National Health and Nutrition Examination Survey Follow-up Study. Arch. Intern. Med..

[42] Xu X., Cheng Y., Li S., Zhu Y., Xu X., Zheng X., Mao Q., Xie L. (2014). Dietary Carrot Consumption and the Risk of Prostate Cancer. Eur. J. Nutr..

[43] Nordström T., van Blarigan E.L., Ngo V., Roy R., Weinberg V., Song X., Simko J., Carroll P.R., Chan J.M., Paris P.L. (2016). Associations between Circulating Carotenoids, Genomic Instability and the Risk of High-Grade Prostate Cancer. Prostate.

[44] Antoni S., Ferlay J., Soerjomataram I., Znaor A., Jemal A., Bray F. (2017). Bladder Cancer Incidence and Mortality: A Global Overview and Recent Trends. Eur. Urol..

[45] Cumberbatch M.G., Rota M., Catto J.W.F., la Vecchia C. (2016). The Role of Tobacco Smoke in Bladder and Kidney Carcinogenesis: A Comparison of Exposures and Meta-Analysis of Incidence and Mortality Risks. Eur. Urol..

[46] Westhoff E., Maria De Oliveira-Neumayer J., Aben K.K., Vrieling A., Kiemeney L.A. (2016). Low Awareness of Risk Factors among Bladder Cancer Survivors: New Evidence and a Literature Overview. Eur. J. Cancer.

[47] Vena J.E., Graham S., Freudenheim J., Marshall J., Zielezny M., Swanson M., Sufrin G. (1992). Diet in the Epidemiology of Bladder Cancer in Western New York. Nutr. Cancer.

[48] Castelao J.E., Yuan J.M., Gago-Dominguez M., Skipper P.L., Tannenbaum S.R., Chan K.K., Watson M.A., Bell D.A., Coetzee G.A., Ross R.K. (2004). Carotenoids/Vitamin C and Smoking-Related Bladder Cancer. Int. J. Cancer.

[49] Brinkman M.T., Karagas M.R., Zens M.S., Schned A., Reulen R.C., Zeegers M.P. (2010). Minerals and Vitamins and the Risk of Bladder Cancer: Results from the New Hampshire Study. Cancer Causes Control.

[50] Wakai K., Takashi M., Okamura K., Yuba H., Suzuki K.I., Murase T., Obata K., Itoh H., Kato T., Kobayashi M. (2000). Foods and Nutrients in Relation to Bladder Cancer Risk: A Case-Control Study in Aichi Prefecture, Central Japan. Nutr. Cancer.

[51] Schabath M.B., Grossman H.B., Delclos G.L., Hernandez L.M., Day R.S., Davis B.R., Lerner S.P., Spitz M.R., Wu X. (2004). Dietary Carotenoids and Genetic Instability Modify Bladder Cancer. J Nutr..

[52] Ros M.M., Bueno-de-Mesquita H.B., Kampman E., Aben K.K.H., Büchner F.L., Jansen E.H.J.M., van Gils C.H., Egevad L., Overvad K., Tjnøneland A. (2012). Plasma Carotenoids and Vitamin C Concentrations and Risk of Urothelial Cell Carcinoma in the European Prospective Investigation into Cancer and Nutrition. Am. J. Clin. Nutr..

[53] Helzlsouer K.J., Comstock G.W., Morris J.S. (1989). Selenium, lycopene, alpha-tocopherol, beta-carotene, retinol, and subsequent bladder cancer. Cancer Res..

[54] Nomura A.M.Y., Lee J., Stemmermann G.N., Franke A.A. (2003). Serum Vitamins and the Subsequent Risk of Bladder Cancer. J. Urol..

[55] Garcia R., Gonzalez C.A., Agudo A., Riboli E. (1999). High Intake of Specific Carotenoids and Flavonoids Does Not Reduce the Risk of Bladder Cancer. Nutr. Cancer.

[56] Risch H.A., David Burch J., Miller A.B., Hill G.B., Steele R., Howe G.R., Burch J.D., Miller A.B., Hill G.B., Steele R. (1988). Dietary Factors and the Incidence of Cancer of the Urinary Bladder. Am. J. Epidemiol..

[57] Bruemmer B., White E., Vaughan T.L., Cheney C.L. (1996). Nutrient Intake in Relation to Bladder Cancer among Middle-Aged Men and Women. Am. J. Epidemiol..

[58] Virtamo J., Edwards B.K., Virtanen M., Taylor P.R., Malila N., Albanes D., Huttunen J.K., Hartman A.M., Ivi Hietanen P., Maè Enpaè Aè H. (2000). Effects of Supplemental Alpha-Tocopherol and Beta-Carotene on Urinary Tract Cancer: Incidence and Mortality in a Controlled Trial (Finland). Cancer Causes Control..

[59] Roswall N., Olsen A., Christensen J., Dragsted L.O., Overvad K., Tjønneland A. (2009). Micronutrient Intake and Risk of Urothelial Carcinoma in a Prospective Danish Cohort. Eur. Urol..

[60] Hotaling J.M., Wright J.L., Pocobelli G., Bhatti P., Porter M.P., White E. (2011). Long-Term Use of Supplemental Vitamins and Minerals Does Not Reduce the Risk of Urothelial Cell Carcinoma of the Bladder in the VITamins And Lifestyle Study. J. Urol..

[61] Xu X., Zhu Y., Ye S., Li S., Xie B., Meng H., Wang S., Xia D. (2021). Association of Dietary Carrot Intake With Bladder Cancer Risk in a Prospective Cohort of 99,650 Individuals With 12.5 Years of Follow-Up. Front. Nutr..

[62] Wu S., Liu Y., Michalek J.E., Mesa R.A., Parma D.L., Rodriguez R., Mansour A.M., Svatek R., Tucker T.C., Ramirez A.G. (2020). Carotenoid Intake and Circulating Carotenoids Are Inversely Associated with the Risk of Bladder Cancer: A Dose-Response Meta-Analysis. Adv. Nutr..

[63] Dianatinasab M., Forozani E., Akbari A., Azmi N., Bastam D., Fararouei M., Wesselius A., Zeegres M.P. (2022). Dietary Patterns and Risk of Bladder Cancer: A Systematic Review and Meta-Analysis. BMC Public Health.

[64] Park S.Y., Ollberding N.J., Woolcott C.G., Wilkens L.R., Henderson B.E., Kolonel L.N. (2013). Fruit and Vegetable Intakes Are Associated with Lower Risk of Bladder Cancer among Women in the Multiethnic Cohort Study. J. Nutr..

[65] Wu J.W., Cross A.J., Baris D., Ward M.H., Karagas M.R., Johnson A., Schwenn M., Cherala S., Colt J.S., Cantor K.P. (2012). Dietary Intake of Meat, Fruits, Vegetables, and Selective Micronutrients and Risk of Bladder Cancer in the New England Region of the United States. Br. J. Cancer.

[66] García-Closas R., García-Closas M., Kogevinas M., Malats N., Silverman D., Serra C., Tardón A., Carrato A., Castaño-Vinyals G., Dosemeci M. (2007). Food, Nutrient and Heterocyclic Amine Intake and the Risk of Bladder Cancer. Eur. J. Cancer.

[67] Hung R.J., Zhang Z.F., Rao J.Y., Pantuck A., Reuter V.E., Heber D., Lu Q.Y. (2006). Protective Effects of Plasma Carotenoids on the Risk of Bladder Cancer. J. Urol..

[68] Holick C.N., de Vivo I., Feskanich D., Giovannucci E., Stampfer M., Michaud D.S. (2005). Intake of Fruits and Vegetables, Carotenoids, Folate, and Vitamins A, C, E and Risk of Bladder Cancer among Women (United States). Cancer Causes Control.

[69] Cairns P. (2011). Renal Cell Carcinoma. Cancer Biomark..

[70] Al-Bayati O., Hasan A., Pruthi D., Kaushik D., Liss M.A. (2019). Systematic Review of Modifiable Risk Factors for Kidney Cancer. Urol. Oncol. Semin. Orig. Investig..

[71] Sahin K., Cross B., Sahin N., Ciccone K., Suleiman S., Osunkoya A.O., Master V., Harris W., Carthon B., Mohammad R. (2015). Lycopene in the Prevention of Renal Cell Cancer in the TSC2 Mutant Eker Rat Model. Arch. Biochem. Biophys..

[72] Bock C.H., Ruterbusch J.J., Holowatyj A.N., Steck S.E., van Dyke A.L., Ho W.J., Cote M.L., Hofmann J.N., Davis F., Graubard B.I. (2018). Renal Cell Carcinoma Risk Associated with Lower Intake of Micronutrients. Cancer Med..

[73] Hu J., la Vecchia C., Negri E., DesMeules M., Mery L. (2009). Canadian Cancer Registries Epidemiology Research Group Dietary Vitamin C, E, and Carotenoid Intake and Risk of Renal Cell Carcinoma. Cancer Causes Control.

[74] Yuan J.-M., Gago-Dominguez M., Castelao J.E., Hankin J.H., Ross R.K., Yu M.C. (1998). Cruciferous Vegetables in Relation to Renal Cell Carcinoma..

[75] Brock K.E., Ke L., Gridley G., Chiu B.C.H., Ershow A.G., Lynch C.F., Graubard B.I., Cantor K.P. (2012). Fruit, Vegetables, Fibre and Micronutrients and Risk of US Renal Cell Carcinoma. Br. J. Nutr..

[76] Bosetti C., Scotti L., Dal Maso L., Talamini R., Montella M., Negri E., Ramazzotti V., Franceschi S., la Vecchia C. (2007). Micronutrients and the Risk of Renal Cell Cancer: A Case-Control Study from Italy. Int. J. Cancer.

[77] Ho W.J., Simon M.S., Yildiz V.O., Shikany J.M., Kato I., Beebe-Dimmer J.L., Cetnar J.P., Bock C.H. (2015). Antioxidant Micronutrients and the Risk of Renal Cell Carcinoma in the Women’s Health Initiative Cohort. Cancer.

[78] Lee J.E., Giovannucci E., Smith-Warner S.A., Spiegelman D., Willett W.C., Curhan G.C. (2006). Intakes of Fruits, Vegetables, Vitamins A, C, and E, and Carotenoids and Risk of Renal Cell Cancer. Cancer Epidemiol. Biomark. Prev..

[79] Bertoia M., Albanes D., Mayne S.T., Männistö S., Virtamo J., Wright M.E. (2010). No Association between Fruit, Vegetables, Antioxidant Nutrients and Risk of Renal Cell Carcinoma. Int. J. Cancer.

[80] Nicodemus K.K., Sweeney C., Folsom A.R. (2004). Evaluation of Dietary, Medical and Lifestyle Risk Factors for Incident Kidney Cancer in Postmenopausal Women. Int. J. Cancer.

[81] van Dijk B.A.C., Schouten L.J., Oosterwijk E., Hulsbergen-Van De Kaa C.A., Kiemeney L.A.L.M., Goldbohm R.A., Schalken J.A., van den Brandt P.A. (2008). Carotenoid and Vitamin Intake, von Hippel-Lindau Gene Mutations and Sporadic Renal Cell Carcinoma. Cancer Causes Control.

[82] Lee J.E., Männistö S., Spiegelman D., Hunter D.J., Bernstein L., van den Brandt P.A., Buring J.E., Cho E., English D.R., Flood A. (2009). Intakes of Fruit, Vegetables, and Carotenoids and Renal Cell Cancer Risk: A Pooled Analysis of 13 Prospective Studies. Cancer Epidemiol. Biomark. Prev..

[83] Zhang S., Jia Z., Yan Z., Yang J. (2017). Consumption of Fruits and Vegetables and Risk of Renal Cell Carcinoma: A Meta-Analysis of Observational Studies. Oncotarget.

